# Are We All Agreed? Consensual Methods and the ‘Necessities of Life’ in the UK Today

**DOI:** 10.1017/S0047279415000033

**Published:** 2015-07

**Authors:** ELDIN FAHMY, EILEEN SUTTON, SIMON PEMBERTON

**Affiliations:** * School for Policy, University of Bristol email: Eldin.Fahmy@bristol.ac.uk; ** School of Oral and Dental Sciences, University of Bristol email: e.sutton@bristol.ac.uk; *** School of Social Policy, University of Birmingham email: s.pemberton.1@bham.ac.uk

## Abstract

In recent decades, consensual approaches to poverty measurement have been widely adopted in large-scale survey research both in the UK and internationally. However, while ascertaining the extent of public agreement on the ‘necessities of life’ has been central to this approach, long-standing critiques have questioned the nature of public consensus on poverty derived using survey methods. By drawing on new primary research preparatory to the *2012 UK Poverty and Social Exclusion Survey*, we consider the contribution of qualitative methods in understanding public views on necessities and discuss their implications for survey-based poverty measurement. Our findings raise some important conceptual and measurement issues for consensual poverty measures within large-scale social surveys. Firstly, our research suggests that public understandings of the term ‘necessity’ are diverse and may not always be consistent with researchers’ interpretations or with wider usage of this term within consensual poverty measurement. Secondly, a better understanding of the considerations which inform survey respondents’ deliberations is needed. Thirdly, our findings have important implications for how we should interpret the concept of ‘consensus’ within the context of consensual poverty surveys, and emphasise the need for the application of more deliberative methods in determining public views on the ‘necessities of life’.

## Introduction

Consensual approaches to poverty measurement reflect widely held concerns about the validity of ‘expert’ judgement as a basis for determining poverty status and have resulted in the development of a range of more participatory approaches to ascertaining minimally adequate household budgets (Bradshaw *et al.*, 2008), incomes (Goedhart *et al.*, 1977; Hagenaars and Van Praag, 1985) and living standards (e.g., Mack and Lansley, 1985; Gordon and Pantazis, 1997; Gordon *et al.*, 2001). Determining the items and activities viewed by the public as constituting ‘necessities of life’ for all households in contemporary society has been at the core of consensual poverty measurement and is our main focus here. This approach purports to extend and develop Townsend's relative deprivation theory in which poverty, arising from insufficient resources, is understood as an enforced inability to participate in lifestyles and activities which are customary or widely approved in contemporary society (e.g., Townsend, 1979, 1987). In particular, a consensual methodology is proposed to ascertain public perceptions of what actually constitutes minimally acceptable living standards and, on this basis, to inform the survey measurement of social and material deprivation.

This methodology therefore seeks to address earlier criticisms of ‘expert’ judgements on the items and activities needed to achieve minimally acceptable living standards by measuring deprivation relative to prevailing public perceptions of these standards. This methodology has been widely adopted in recent decades, both in the UK and elsewhere, in order to better understand the extent and social distribution of poverty vulnerability. Nevertheless, as Walker (1987) has long argued, the nature of the ‘consensus’ generated using consensual survey methods is not currently well understood, and existing evidence derived using these methods reveals relatively little about the nature of public understandings of ‘necessities’ and the considerations that guide public deliberations on specific items and activities. Indeed, although Walker was responding to the ground-breaking work of Mack and Lansley (1985) more than a quarter of a century ago, surprisingly little has changed in the intervening period. Existing work in this area using survey and qualitative methods has focused heavily upon the outcomes of deliberation (i.e., lists of necessities items), largely at the expense of engaging with the content of the deliberative process itself which might facilitate better understanding of the nature of the ‘consensus’ reached. In this paper, we therefore reflect upon the applications of qualitative methods in advancing consensual approaches to poverty measurement in Britain today in order to begin to address this gap in knowledge. We begin by discussing the implementation of consensual poverty measures as operationalised within the UK ‘breadline’ surveys, and review existing qualitative evidence on public understandings of what constitutes minimally acceptable living standards. On the basis of qualitative development work preparatory to the *2012 UK Poverty and Social Exclusion Survey* (2012 PSE-UK), we then outline some key conceptual and measurement issues in determining the ‘necessities of life’ and consider their wider implications for survey-based consensual poverty measurement.

## Survey evidence on poverty and the necessities of life

In recent decades, consensual approaches to poverty measurement have been widely adopted in large-scale survey research both in the UK and internationally. This approach was pioneered in the UK in the ‘breadline’ series of poverty surveys as originally implemented in the *1983 Poor Britain* survey (Mack and Lansley, 1985) and developed and refined in subsequent surveys in Britain (Gordon and Pantazis, 1997; Gordon *et al.*, 2001) and Northern Ireland (Hillyard *et al.*, 2003). It is also the approach taken to poverty measurement in the 2012 PSE-UK survey. In recent years, this approach has also been more widely adopted internationally in order to better measure living standards and social and material deprivation. This includes across the European Union as a whole (Guio *et al.*, 2012) and in Sweden (Hallerod, 1995, 1998), Finland (Kangas and Ritakallio, 1998), Ireland (Nolan and Whelan, 1996; Layte *et al.*, 1999), Belgium (van den Bosch, 1998), and The Netherlands (Muffels, 1993). The approach is also increasingly widely applied further afield in both high-income countries such as Australia (Saunders, 2011; Saunders and Wong, 2011), Japan (Abe, 2010), Russia (Tchernina, 1996), New Zealand (Perry, 2009), and in middle- and low-income countries including Bangladesh (Ahmed, 2007), South Africa (Wright, 2011) and Vietnam (Davies and Smith, 1998). Although the primary research on which this paper is based focuses on the application of the consensual poverty methods in the UK, we believe that the issues it raises have much wider relevance for international poverty-research practice.

Conceptually, the consensual approach has its roots in Townsend's relative deprivation theory of poverty (e.g., Townsend, 1979, 1987). From this perspective, poverty is viewed as an insufficient command of resources over time resulting in an inability to fulfil needs (i.e. deprivation). Crucially, needs are here understood as socially determined and relative to prevailing normative standards. However, in response to long-standing critiques of the limitations of expert judgement in determining the ‘necessities of life’, social survey methods have since the *1983 Poor Britain* study (Mack and Lansley, 1985) been used to ascertain the public's views on what constitutes contemporary necessities. These public judgements have been incorporated into the subsequent survey measurement of the extent of deprivation of contemporary necessities.

The most recent operationalisation of this approach is the *2012 UK Poverty and Social Exclusion Survey* (2012 PSE-UK) although the same measurement approach was used in Britain in 1999 (see Gordon *et al.*, 2001) and in Northern Ireland in 2002/03 (see Hillyard et al., 2003), subject to some changes in specific necessities items and their wording arising from the qualitative development work reported here. In the 2012 PSE-UK study, a module on public perceptions of necessities was included in the *Summer 2012 ONS Opinions Survey* in Britain, and in the *June 2012 NISRA Omnibus Survey* in Northern Ireland (Gordon, 2012). Based upon stratified random sampling methods, representative samples of the UK public were asked to determine those items and activities *‘which all adults should be able to afford and which they should not have to do without’* (Gordon, 2012). Using sort card methods, respondents were invited to sort items and activities considered ‘necessary’ and those considered ‘desirable but not necessary'. In consensual approaches to poverty measurement, a public ‘consensus’ on the necessities of life is then said to exist where, on the basis of a representative sample survey design, a simple majority of respondents agree that specified items and activities are ‘necessities’ as defined above *and* that there are no significant social differences in respondents’ perceptions of these items, i.e. that this consensus exists across social groups. The subset of items and activities meeting these criteria can then be used to establish how many households lack these items because they cannot afford them and, subsequently, to derive a consensual deprivation index on this basis.

If, as is now widely accepted, poverty should be conceptualised and measured relative to prevailing normative standards, then there is much to recommend approaches which solicit public views on the ‘necessities of life’ and incorporate these within poverty measurement. However, as we shall see, the extent and nature of public consensus on the meaning of poverty is far from clear. In particular, there are significant technical and conceptual issues in interpreting the nature of public consensus derived using this approach. In the remainder of this paper, we will consider how qualitative evidence can inform understanding of public views on the nature and extent of consensus on the items and activities which constitute the necessities of life in the UK today.

## Consensus by coincidence?

While the theoretical rationale for the consensual approach is now well-established, ascertaining public attitudes towards the contemporary necessities of life is considerably less straightforward than might at first appear. Although the contention, that widespread public consensus exists on the items and activities needed to avoid poverty in our society today, has been central to the consensual approach, the nature and meaning of ‘consensus’ here is currently not well understood. Critics of the consensual approach have raised various concerns about the nature of the ‘consensus’ achieved in sample surveys of perceptions of necessities, for example by highlighting the conceptual and methodological difficulties in establishing a ‘valid’ consensus on the basis of individualised survey responses. As Walker (1987) has argued, existing evidence derived using these methods reveals relatively little about the nature of public understandings of ‘necessities’ and the considerations that guide public deliberations on specific items. Moreover, as this author notes, respondents in surveys on this topic are ‘asked to provide immediate responses to tightly worded questions about complex and sensitive issues to which few of them will previously have given much thought’ (Walker, 1987: 213–214). Related to this prescient observation, Walker goes on to argue that survey evidence on public perceptions of necessities provides no information on the stability of respondents’ views ‒ for example, in the light of empirical evidence on the extent of deprivation, or in the face of wider public discussion of these issues.

As Walker (1987) notes, survey methods tell us little or nothing about the evaluative criteria respondents employ in determining whether specific items are ‘necessary’, or indeed whether the concept of ‘necessity’ is itself equated by respondents with those items which ‘no-one should have to do without’ as generally intended by researchers adopting consensual approaches. Walker's observations (1987: 219, 221) led him to describe the consensual approach as originally operationalised by Mack and Lansley (1985) as representing ‘consensus by coincidence’, and to conclude that their own consensual survey methods:

are unable to say anything about the criteria which people employ in judging whether or not items are ‘necessary’ nor, indeed, whether respondents felt equally strongly about each of the items assessed. Is the concept of ‘necessary’ really juxtaposed in people's minds with the notion ‘should not have to do without’ (which would seem to postulate some form of intervention) as the Mack and Lansley question implies? What reference groups do people use? How far are judgements grounded in experience or hearsay? How stable are people's responses in the light of information about living standards and on hearing the views of others?

Although these fundamental issues were first raised more than a quarter of a century ago, they remain pressing questions in evaluating the adequacy of existing approaches to consensual poverty measurement and in interpreting subsequent results.

While consensual poverty surveys in Britain in 1999 and 2012 demonstrate a relatively high degree of social homogeneity in public perceptions of necessities (e.g., Pantazis *et al.*, 2006; Mack *et al.*, 2013; though see also McKay, 2004), it is clear that the meaning of poverty itself varies widely among the general public (see ‘Qualitative evidence on poverty and the necessities of life’ below). In addition, as McKay (2004) argues on the basis of analysis of the 1999 Poverty and Social Exclusion Survey of Britain (Gordon *et al.*, 2002) dataset, there is substantial variation between individual respondents in their assessments of items and activities. Existing survey work in this area typically defines ‘consensus’ operationally in terms of majority support *and* between-group agreement on the items and activities which no-one should have to go without. However, regardless of the extent of between-group effects relating to socio-economic or demographic distinctions, the degree of inter-rater agreement on necessities is quite low, suggesting that individual respondents classify quite different sets of items and activities as ‘necessities’. Thus, the relative absence of social differences in evaluations of specific items may not necessarily imply widespread public agreement on the set of items and activities which all households should be able to afford.

Nevertheless, the sources of inter-personal variation in perceptions of necessities have been subject to little scrutiny. Here we argue that qualitative evidence can make an important contribution in advancing understanding of these issues. Our findings suggest that public understandings of the term ‘necessity’ are diverse and may not always be consistent with researchers’ interpretations, or with current usage in survey-based measurement. These findings have important implications for how we interpret ‘consensus’ within survey-based consensual poverty measures and, more widely, for our understanding of the potential offered by qualitative evidence in informing the design and conduct of survey instruments. Our main focus here is on the conceptualisation of consensus itself and the methods deployed to estimate and interpret the degree of public consensus on the ‘necessities of life’ in the UK today. We begin contextualising this discussion by briefly reviewing existing qualitative work on public perceptions of poverty and necessities in the UK.

## Qualitative evidence on poverty and the necessities of life

Since the 1990s, an emphasis upon inclusive approaches using participatory methods has considerably advanced understanding of the nature and meaning of poverty and its material, social and psychological impacts, including the perspectives of the ‘real experts’, people experiencing poverty. Such studies do not, in general, provide clear empirical support for the existence of a public consensus regarding the meaning and definition of poverty. Rather, they demonstrate the *plurality* of public conceptions of poverty. For example, based upon discussion groups with low-income citizens Beresford *et al.* (1999) found that, while a consensus existed around an irreducible ‘absolute’ interpretation based on physiological functioning, views differed markedly on broader definitions of poverty based upon relative deprivation of material and social needs. As part of work preparatory to the 1999 GB-PSE, Middleton (1998) conducted focus groups to explore perceptions of the necessities of life. While these authors focus mostly on specific items and activities, their findings suggest substantial variations may exist in public perceptions of the nature of poverty ranging from absolute definitions, based upon physiological need, to relative accounts, emphasising social norms and functioning, though these themes are not explored in depth. Participatory research with women across Britain by the Women's Budget Group (2008) similarly suggests considerable plurality in public understandings of poverty that emphasise financial/material constraint and the relational dimensions of poverty (e.g., isolation, stigma, denial of rights, restricted social functioning).

A tendency for low-income participants to define poverty in absolute terms in ways which sometimes discount their own experiences of deprivation is a recurring theme. Flaherty's (2008) research with low-income participants demonstrates how people experiencing poverty sometimes tailor their expectations in ways which underplay their own experiences of deprivation. Similarly, research with older people by Dominy and Kempson (2006) suggests that personal financial circumstances influence perceptions of poverty with better-off participants tending to adopt broader understandings of poverty (e.g., associated with financial security and social participation) compared with low-income participants who tended to view poverty in more restrictive terms associated with extreme forms of marginalisation (homelessness, hunger, etc.), or poverty in low-income countries (see also Crowley and Vuillamy, 2007; Save the Children, 2011). In line with wider qualitative evidence on the existence of adaptive preferences (e.g., Smith *et al.*, 2004; Scharf *et al.*, 2006), these findings suggest that a tendency to discount personal experiences of poverty may be a strategy for coping with the harsh realities of life on a low income. Flaherty (2008) also documents how low-income participants sometimes adopt minimalist definitions of poverty as a discursive strategy for distancing themselves from the stigma attached to poverty. Interestingly, these findings seem to contradict large-scale survey evidence which suggests that poor households often have a more generous interpretation of the necessities of life (e.g., Pantazis *et al.*, 2006).

In recent years, several studies have adopted qualitative and participatory methods to develop lay consensual approaches to budget setting. Focus-group methods have been widely used, in conjunction with broader consensual budget standards approaches, in order to investigate public perceptions of what constitutes a ‘minimally acceptable’ living standard and to deliberate upon the household budget needed by households of different types to achieve this standard (e.g., Bradshaw *et al.*, 2008; Hirsch *et al.*, 2009; Hirsch and Smith, 2010; Davis *et al.*, 2010). These studies demonstrate the challenges involved in investigating public views on the items and activities considered to be necessities of life in Britain today. Indeed, as Hirsch and Smith (2010) note, participants sometimes experience difficulty in evaluating whether deprivation of specific items constitutes ‘identifiable hardship’ in the absence of contextual information on the wider basket of goods, services and activities available to households. Often participants’ deliberations appear to involve subtle distinctions between items and activities which are normatively valued and those which it is ‘harmful’ to lack, especially with regard to the long-term negative consequences for individuals and families.

Qualitative research with specific population groups also demonstrates the extent to which public perceptions of necessities depend upon contextual information about the composition and circumstances of households such that no single basket of items and activities is likely to be adequate in characterising adequate living standards for all households. A specific focus on different populations reveals quite different sets of preferences regarding the necessities of life, for example, for disabled people (Smith *et al.*, 2004), families with children (Middleton *et al.*, 1994; Hirsch and Smith, 2010) and households in rural areas (Smith *et al.*, 2010). Although these studies adopt a consensual budget standards methodology, which differs somewhat in its overall objectives from the socially perceived necessities approach, they again draw attention to the importance of contextual information about households’ circumstances when making public judgements concerning the items and activities which all people need in order to achieve minimally adequate living standards in our society today.

A further point of contention involves considerations of *quality*, *intensity* and *frequency* in determining the items and activities which all households should be able to have and do. For example, while the importance of social relationships and participation is often prominent in participants’ accounts, as Hirsch and Smith (2010) note, it is much more difficult to establish consensus upon *how much* social participation is necessary with regard to considerations of duration, intensity and frequency of participation. Similarly, qualitative distinctions regarding the quality of goods and services are often decisive in public deliberations on necessities but are also highly complex, involving subtle social distinctions associated with changing tastes, norms and lifestyles. As Bradshaw *et al.* (2008) argue, this suggests that the capacity for some degree of *choice* in making consumption decisions is an important factor in group discussions of the items necessary to achieve minimally acceptable living standards with regard to, for example, diet, clothing, leisure activities, etc. Nevertheless, it remains unclear to what extent this perspective can be accommodated within survey-based measurement of public perceptions of necessities.

## Determining the necessities of life in the UK today: qualitative evidence

In this paper, we illustrate some of the above issues by drawing on the qualitative development work preparatory to the *2012 UK Poverty and Social Exclusion Survey* using focus-group methods. A total of fourteen focus group interviews were conducted in late 2010 in Bristol, Cardiff, London, Glasgow and Belfast with a total of 114 participants. Separate group interviews were conducted with low-income, non-low-income and mixed-income samples, and groups were also stratified by household type, and minority ethnic status.^1^ Prior to attending the group discussions, participants were asked to complete a recruitment survey (collecting basic participant socio-demographic data) and a brief open-format questionnaire on the ‘necessities of life’ and social exclusion in the UK today, to encourage participants to begin to think in advance about these issues.

Research was conducted in two overlapping phases. In Phase One, focus-group participants were asked to suggest necessities items in a relatively unstructured way using brainstorming methods. Participants were asked to deliberate upon those items and activities which they considered to be necessities for a ‘typical’ family with children in the UK today, based upon the situation of a hypothetical family comprising a married, single-earner couple with two children aged eight and twelve living in the suburbs of a large city. Drawing on these suggestions, alongside some additional living standards’ items derived from earlier UK studies (i.e., including items *not* widely viewed as necessities in earlier surveys), participants in Phase Two were then invited to deliberate on whether these items constitute ‘necessities of life’ in relation to the circumstances of the same ‘typical’ family. In order to focus thinking on these issues, participants were invited to reflect in an unstructured way on potential items and activities they considered important, in documentation issued prior to attending the group itself.

Our expectation was that a wider public consensus may exist where Phase 2 groups independently classified broadly the same subset of items and activities as ‘necessities’ as those initially suggested in the more exploratory Phase 1 groups. It should be noted that the dynamics of participant interactions within focus groups tend towards consensus through the interrogation of inter-subjectivities arising from the dynamics of group dialogue (e.g., Kreuger and Casey, 2009; Stewart *et al.*, 2007). It is difficult to make definitive statements concerning the impact of such forms of ‘collective reasoning’ on the selection of items. However, it is instructive to look at how the process of deliberation operates in achieving consensus within a group discussion context, and at how these processes may differ from the response process undertaken by individual survey respondents in selecting items within a household social survey. In practice, group decisions on many items were often made on the basis of universal or near-universal agreement and, where strong differences of opinion existed, a majority decision was recorded. Here, we explore this deliberative process by examining participants’ decision-making strategies in the qualitative group discussions and the light this can shed on the nature of the survey task, for example, with regard to issues of cognition, judgement, recall and sensitivity. Our focus here is upon the factors informing individual participants’ decision making rather than the dynamics of group interactions *per se*. However, as noted above, the dynamics of group discussions tend towards consensus and this effect may have moderated the public expression of more ‘extreme’ views amongst participants advocating minimalist and maximalist interpretations of contemporary necessities.

### Definitional issues

All groups were invited to reflect upon how poverty is best characterised and understood in order to probe participants’ perspectives on the nature and meaning of poverty. While it is difficult to fully convey the diversity and complexity of these accounts here, it was evident that very different views existed on the nature and meaning of poverty, and that participants’ views were not always stable but rather that their perspectives tended to broaden as discussion developed. In line with previous studies (e.g., Crowley and Vulliamy, 2007; Flaherty, 2008), many participants initially suggested restrictive ‘absolute’ definitions of poverty by citing examples of extreme poverty and marginalisation both in low-income counties and in the UK. However, this did not necessarily imply a rejection of ‘relative’ accounts of poverty:

GLS1 RM: When I think of poverty, I do think absolute poverty . . . that's the thing that pops up into my head, rather than poverty which I know that there is over here.

LDN2 RF: Poverty's relative to the country you’re living in. What is called [poverty] in the UK today might be a wealthy environment for somebody from another part of the world.

GLS1 RM: Everything's relative to circumstances around you. I mean you could be living in the very richest suburb and be the poorest person in that neighbourhood, think of yourself as poor, when by national standards you’re nowhere near it. And you could be living in the poorest area but because . . . you got a job, you could be the richest person there.

BRS2 RM: There's a big envelope if you’ve got someone on the street who's got nothing, they’re in poverty, you’ve got someone who's living in council accommodation with a couple of kids, just got a few pennies to scrape by, you could say they’re in poverty.

Often, subsequent discussion led to a considerable broadening of perspective as participants considered the adverse impacts of insufficient resources on social participation, networks and support, housing and living conditions, health, quality-of-life and well-being. Participants emphasised the social dimensions of poverty with regard to the fulfilment of social roles and normative expectations as well as its psycho-social impacts. At the same time, participants often also defined poverty as having to forego items and activities which are commonly taken for granted by people living in the UK today:

NI3 RF: Living on the breadline is poverty to me, just things being very tight and just barely keeping your head above water . . . just necessities rather than luxuries.

CDF2 RM1: Living on the breadline. You’ve just got the bare necessities; no luxuries. That's it, just getting through.

LDN3 RF: Where you don't meet the basic needs of common living . . . you’re worried where is the next meal coming from.

CDF3 RF2: Not being able to make ends meet by doing things that you should really be able to afford to do every day.

The above quotes might suggest that participants’ perspectives were largely consistent with the general approach to poverty measurement adopted within the ‘breadline’ studies. Nevertheless, participants’ perspectives were complex and multi-faceted so that no single operational definition would be likely to accommodate these nuances of meaning. This interpretation was reinforced in subsequent group discussions in which participants were invited to comment on the appropriateness of subsistence, basic needs and relative definitions of poverty.^2^

In general, definitions of subsistence and basic needs were most easily understood and most readily elicited widespread agreement (i.e., consensus) among participants. However, many participants emphasised the social nature of needs, though sometimes with very different implications in terms of the kind and range of items and activities felt to be necessities in the UK today.

While the terminology of need and necessity was often used by participants, the ambiguities of meaning associated with these terms were also clear. In some instances, necessities were defined with reference to contemporary living standards and consumption norms or by referring to the social significance of items aside from their functional utility. To this extent, ‘need’ is socially constructed in relation to participants’ perceptions of prevailing norms within contemporary society. Consideration of what is ‘reasonable’ or ‘adequate’ was central to participants’ decision-making reflecting social judgements relating to norms of self-presentation, the avoidance of shame and the value of social connections and norms. Nevertheless, while the social pressure to ‘keep up’ with contemporary patterns of consumption (however extravagant) was acknowledged to create the potential for new social distinctions and exclusionary processes to emerge, such items were not always considered as ‘necessities’. Many participants’ accounts of their decision-making referred to estimations of how difficult it would be to do without specific items, and therefore of the extent to which items and activities may be seen as ‘luxuries’, regardless of how inexpensive or how widely enjoyed such items may be:

LDN3 RM: Is it really a necessity to have a DVD player? It's not about the price because if something is cheap it doesn't mean you should go out and buy it. But if it's available and you can definitely do without it, then I don't see it as a desirable or a necessity but as a luxury

BRS2 RF: Not having the basic necessities of life . . . There's different extremes of not being able to have like the latest technology and that sort of thing, it's not necessarily poverty, you might be excluded from a certain society but you’ve still got the basic things

LDN1 RF1: I struggle with people's definitions of what a luxury is and necessities, and there's certain objects for me like Wii or DVD players . . . people now feel that these things are necessities and, you know, basics that they should have, they think that they need to have that an item which is not exactly very key to their sort of core life, and that's why I say that certain objects are luxuries

As demonstrated by the above quotes, while acknowledging the social pressures driving consumption, some participants distinguished between items and activities perceived to be necessary to human flourishing and those perceived to be driven by the consumer society in which we live. Thus, expert definitions receive a mixed reception here, with their legitimacy contested in relation to items sometimes perceived to be driven by mass consumerism rather than the satisfaction of basic needs.

### Cognition issues

Our qualitative evidence suggests that the survey task faced by respondents is both conceptually complex and subject to numerous ambiguities in terms of the interpretation of question wording. For example, some focus-group participants interpreted the task as requiring an assessment of those items that people would be likely to prioritise even if they were experiencing poverty, rather than the items all people should not have to go without (i.e., in a normative sense). In other words, ‘necessities’ here are taken to mean those items remaining once all non-essential spending has been cut from the budgets of low-income households. Other participants’ judgements of specific items implied an evaluation of whether specific items are important in avoiding poverty in a definitional sense. For example, when asked if all adults should be able to afford some new clothes, one participant remarked:

BRS1 RM: I’m going to say no. I don't think having new clothes is what takes you out of poverty personally.

BRS1 RM: I don't doubt that *[lots of people prefer new*] but I don't think that's what we’re asking, I don't think it's a preference issue, I think it's . . . is that poverty? And I don't think it is, because I think you can have anything if you actually put your mind to it

In some groups, the terminology used in consensual research methods was questioned, with differences of opinion emerging between participants on the meaning of the term ‘necessity’ itself. Some participants expressed concerns about the interpretation of ‘necessity’ to denote items or activities that all people should be able to afford, rather than to denote those items and activities people simply *cannot* live without (e.g. in the quote below, when referring to meat and fish as dietary necessities). To this extent, researchers’ interpretations of the term ‘necessity’ may not always be consistent with wider public understandings of the term which frequently referred to items and activities which were viewed as impossible to go without, implying a more restrictive interpretation of need. Despite prompts from the researcher, some participants understood the task to involve selecting items necessary for people experiencing poverty rather than those items needed to avoid it:

BRS2 RM: Not a necessity, no . . . In an ideal world, yeah, everyone loves a bit of meat and a bit of fish and some, but surely if you’re on the poverty line a bowl of porridge would just see you through.

GLS1 RM: There's a difference between what that family should be able to afford and what a necessity is . . . Maybe changing necessity to affordability, I think that's the word you’re missing. I would say a TV is absolutely 100% this family should be able to afford, but it's not a necessity so it's difficult.

NI3 RF: Well the way I would have to look at necessity is can you survive without it

Other participants made distinctions between an item's economic costs and its social benefits in ways which again draw attention to the normative dimensions of the interpretation of necessities within consensual approaches to poverty measurement:

GLS1 RM: Because if we’re asking the question do we think this family should be able to afford it, then that's an economic question. Do I think it's socially beneficial for them to have this? Then yes.

To this extent, references to what households and individuals ‘should’ be able to afford are potentially ambiguous in referring both to a normative judgement about entitlements, as well as to evaluative judgements concerning what households and individuals are in fact likely to be able to afford and need. The latter interpretation of ‘necessities’ as items that are both affordable and widely enjoyed, and also impossible to do without in our society today, was one widely supported within these discussion groups. Again this is not necessarily consistent with the use of this term by social researchers, though it could be argued that such an interpretation is, in principle, consistent with Townsend's relative deprivation theory of poverty which underpins consensual approaches (a point to which we will return below).

### Need, entitlement and the abstract individual

Our qualitative evidence suggests that the survey task presented to respondents may lack sufficient contextual information for respondents to provide meaningful responses. Although the survey task refers to items and activities which ‘*all* people should be able to afford’, it quickly became apparent in the piloting of the focus group instruments and in their subsequent application that many participants felt they lacked the necessary contextual information to make a reasoned decision on necessities items on this basis. In the conduct of the focus groups, participants were provided with a hypothetical scenario or ‘vignette’ to facilitate group decision-making on ‘necessities of life’ items.

Nevertheless, some participants had difficulty in making judgements on whether specific items should be viewed as necessities in the absence of further contextual information which might aid their decision-making processes, such as the family's level of income, or issues related to the contemporary costs of living for households in different circumstances. For example, in evaluating whether access to a car should be classified as a ‘necessity’, many participants felt they required information on the availability of affordable public transport, household composition (especially in relation to children and elderly or disabled residents), geographical location, working patterns and so on. Related to this point, such decisions also appeared to be difficult for some participants in the absence of further information on the full basket of goods, activities and services available to households and the extent to which different items may be substitutable (i.e., car *vs* affordable public transport; internet access *vs* telephone, etc.):

GLS2RF: It depends how much he's earning first and foremost . . . it just really depends.

LDN2 RM: What sort of accommodation would he be able to afford? Are they social housing, are they private housing?

BRS1 RM: I’d say a car only if public transport not available.

NI1 RM: I think it depends where you work and where your schools are.

Such contextual information was also perceived to be important in shaping participants’ normative judgements concerning entitlements, often based upon underlying moral distinctions between the ‘deserving’ and ‘undeserving’ poor. Reference was made in many groups to the entitlements that were perceived to arise from fulfilment of social roles as workers and as parents. There were, however, differing opinions expressed on issues of eligibility for people living on a low income or who were perceived to be ‘welfare-dependent’. While some referred to notions of universal entitlement, others made distinctions between the ‘working’ population and the ‘poor’, and sometimes between what we expect for ourselves and for others:

LDN3 RM: If you’re saying there's nobody working in the house then I’d say no way, but if you’ve got a working household you would hope in this country that people could [*go out for a meal*].

CDF2 RF: I’m not being horrible to poor people but why should they be allowed to have double glazing when people who are working can't afford it.

## Discussion: key issues with survey-based consensual measures

What, then, can we conclude regarding the contribution of qualitative methods in informing understanding of public perceptions of the necessities of life and of the definition and meaning of poverty more generally? The consensual measurement framework is now well-established in poverty-research practice, and survey methods have been central to efforts to establish the extent of social consensus on the ‘necessities of life’ in the UK for more than twenty-five years. Nevertheless, little attention has focused on the nature of the consensus generated using survey research methods, for example, by interrogating respondents’ decision-making processes in reaching judgements on necessities items, in understanding respondents’ own views on the concept of ‘necessity’ itself or in considering the stability of respondents’ views on these items. Although focus-group methods were employed in the development of question items on the necessities of life, both in Mack and Lansley's (1985) ground-breaking study *Poor Britain* and in the 1999 PSE-GB survey (to date the most comprehensive survey of its kind ever undertaken in the UK) (see Middleton, 1998), little serious attention has been focused on these basic conceptual issues.

Firstly, the concept of ‘consensus’ itself is under-theorised in understanding public perceptions of minimally adequate living standards. Consensus is a multi-faceted concept referring both to ‘general agreement’ and a ‘majority view’. Drawing upon Martinez-Panero's (2011) analysis, consensual poverty measurement appears to draw upon Condorcet's formalisation of Rousseau’s theory of the ‘general will’, positing the existence a common good which individuals may not be able to accurately ascertain but which can be reliably estimated on the basis of public deliberation, and results in a form of agreement which crucially represents more than simply the aggregation of individual preferences. It is this broad conception of consensus that has informed both deliberative conceptions of justice as reflected in the work of Rawls (1971/1999) and Habermas (1984), and social choice theories (e.g., Arrow, 1951/1978; Riker, 1982).^3^

While a consideration of these broader arguments is beyond the scope of this article, their implications for consensual approaches to poverty measurement certainly requires further theoretical enquiry.

Secondly, as the above discussion illustrates, there are compelling grounds for questioning the extent to which existing survey approaches actually provide solid evidence of widespread public agreement on the necessities of life. In particular, it is unclear whether the nature of the task required of survey respondents is capable of yielding informed deliberations on the complex question of ‘what are the necessities of life’. In the qualitative development work reported here, the use of pre-group materials was an effective means of stimulating thinking prior to the group interaction itself and this frequently led to a reconsideration of participants’ views in the light of subsequent group discussion. While the reactivity this approach implies would generally be considered inappropriate in a survey context, the absence of stability in participants’ views nevertheless illustrates the complexities involved in public judgements on these issues. In contrast, survey respondents are typically tasked with providing immediate responses in relation to these complex issues, which may be unfamiliar for many participants. Participants are asked to focus upon very specific items and to make snap judgements, using the response categories provided by experts, without any contextual information on the circumstances of households or the other goods and activities available to them which might usefully inform their deliberations.

As a result, the nature of the consensus generated using these methods remains uncertain. We have noted that existing analyses of survey data for the 1999 PSE-GB reveal few signs of significant between-group variations (Pantazis *et al.*, 2006), but that the degree of inter-rater agreement is comparatively low (McKay, 2004). Given the evidence reviewed above, it is quite plausible that these apparently random variations may, in large part, reflect substantial differences between individual survey respondents in their interpretation of the required task. To this extent, any ‘consensus’ disguises considerable diversity in the rationales underpinning support for specific necessities items. Since survey methods by their nature provide no information on the viewpoints which guide respondents’ deliberations, they are also uninformative in explaining the dynamics of public views on these issues. Researchers thus remain unable to comment on the dynamic and fluid social contexts that attribute meaning to ‘necessity’ items, in the process restricting analysis to description without offering meaningful interpretation of survey findings. Moreover, in line with existing qualitative work in this area, the above findings emphasise the plurality of public conceptions of poverty, as well as the sometimes unstable and contradictory nature of participants’ views. These findings suggest that, while the general framework proposed by advocates of the socially perceived necessities approach may well be consistent with public understandings of poverty, this does not imply widespread agreement on specific items and activities which all people should be able to afford and should not have to go without.

As has already been noted, in contrast with survey methods, the dynamics of focus-group interactions tend towards consensus as a result of the sharing of ideas and experiences. Nevertheless, even in this context, widespread agreement on specific items and activities proposed by participants was sometimes difficult to achieve and decisions, in some cases, were reached on the basis of a majority verdict. In the context of the survey measurement of public attitudes towards the ‘necessities of life’, a ‘majoritarian’ methodology (rather than a consensual one) may be a more accurate description of the overall approach. Assessing the extent to which such findings are method-dependent is a pressing task given the obvious differences in approach and context in ascertaining public perceptions of necessities between qualitative and survey-based methods. To our knowledge, existing work in this area has not, to date, been informed by more rigorous approaches to the testing and development of survey items. However, given the evident variability in participants’ interpretations of the required task, further question testing using methods such as cognitive testing and behaviour coding is certainly warranted.

More generally, given the overall thrust of the consensual approach, these findings also emphasise the importance of taking participants’ perspectives more seriously in the context of qualitative work on the necessities of life if we are to fully realise the potential of consensual methods in the measurement of poverty. As Walker (1987) rightly concluded more than twenty-five years ago:

To be true to the consensual approach, people must be given scope to express their views. They need time to find their own words, to reflect on their own experience, and to grapple with the complexities of the subject. Researchers must equally be prepared to listen to their respondents and to work with their ‘real-world’ concepts. Similarly they should be willing to enter into a dialogue with their respondents. Opinions grounded in ignorance, while interesting in themselves and sometimes valuable as predictors of behaviour, have little utility as a basis for policy not least because they are likely to be very unstable. Moreover they do not do justice to the intellect of the respondents or to their presumed commitment to the research exercise. Researchers are therefore obliged to provide respondents with the information which they need in order to make reasoned choices and, as far as possible, to provide feedback on the consequences of the choices made (Walker, 1987: 221)

In recent years, more ‘deliberative’ methods have been increasingly adopted in order to solicit the public's reasoned and considered views on normative questions relating to poverty, income adequacy and well-being. Some important work has since been achieved in this respect, for example, in the development of consensual budget standards in order to ascertain the income needed for different households to meet minimally acceptable living standards (Bradshaw *et al.*, 2008; Hirsch *et al.*, 2009; Hirsch and Smith, 2010; Davis *et al.*, 2010), and in operationalising notions of capability and well-being (Burchardt and Vizard, 2009, 2011). Nevertheless, the application of deliberative methods in advancing understanding of public views on ‘the necessities of life’ remains an under-researched topic and a pressing priority in relation to consensual poverty measurement approaches.

Such methods can potentially augment the ‘scientific’ credentials of subsequent survey-based poverty measurement by illuminating processes of public deliberation and private choices on normative questions relating to minimally adequate living standards. However, as Burchardt (2014) notes, while deliberative methods are a well-established technique within the context of public decision-making and citizen participation (e.g., Stewart, 1995; Fishkin, 1997, 2009), as a research technique they remain substantially under-theorised. Whether genuine intersubjective consensus on such contentious, normative issues is ever possible in deeply divided societies is itself questionable (e.g., Young, 2000; Wakeford, 2008), but more pragmatic questions concerning the authenticity of deliberative methods as a research practice also need to be considered, for example, relating to:

[T]he way topics for deliberative research should be identified; the function of expertise and the nature of evidence; the nature of public reasoning in non-ideal situations; the role that language plays in expressing power relations among participants and between participants, facilitators and experts; the status of people's pre- and post-deliberation views and opinions and the analysis of potentially conflicting results (Burchardt, 2014: 365)

These observations certainly raise some fundamental questions about the extent to which expert and experiential knowledge can be genuinely reconciled in deliberative research on normative issues concerning minimally acceptable living standards. However, notwithstanding these important methodological concerns, we believe that the research described here sheds some light on the processes of deliberation and the criteria guiding public judgements on poverty. In particular, the research problematises public understandings of the term ‘necessity’ itself and how this relates to the concept of poverty and its measurement. On the basis of these findings, the extent to which an unambiguous consensus in fact exists on the ‘necessities of life’ certainly requires further scrutiny. These findings illustrate the ways in which decisions on these issues are shaped by dialogue and by the contextual information available to participants. Moreover, the extent to which the necessities framework is itself considered by the public as the most appropriate way of conceptualising and measuring poverty is very much open to question. These are vitally important issues, which consensual poverty measurement will need to more fully engage with in future if such methods are to fully live up to their self-description.

## Notes

1 Respondents were recruited by professional recruitment consultants according to a recruitment plan provided by the research team with no prior screening. ‘Low income’ in this context referred to households with incomes of less than 60% of median equivalised incomes by household type (based upon 2011 HBAI statistics). Separate groups were conducted with pensioners, single parents, couples with children, and working age adults without dependent children, as well as with ethnic minority participants. Most groups comprised eight to ten participants and were of approx. 2.5 hours in total duration. All participants received a one-off gift payment of £35 plus travel expenses in recognition for their contribution to the research.

2 Participants were asked to deliberate on the following statements: ‘Households living in the UK today are poor if. . .’: (a) ‘They do not have sufficient resources to meet their physical needs for food, shelter, warmth, light and sanitation for all members of the household’ (subsistence); (b) ‘They do not have sufficient resources to meet their physical needs and lack adequate access to education, information, and health and social care for all members of the household’, and; (c) ‘They do not have sufficient resources to fully participate in activities and living patterns which are widely available in the UK today’ (relative poverty)

3 The literature on deliberative justice is extensive. See Elster (1998), Fishkin (2009), and Goodin (2003) for a review of these ideas and their origins.
